# Exploring delay to diagnosis of endometriosis, a healthcare professional perspective

**DOI:** 10.1186/s12913-025-13536-5

**Published:** 2025-11-18

**Authors:** Babu Karavadra, Gabrielle Thorpe, Edward Morris, Joanna Semlyen

**Affiliations:** 1 Norwich Medical School, Norwich Research Park, University of East Anglia, Norwich, NR4 7TJ UK; 2 University of Liverpool, Liverpool, UK; 3 School of Health Sciences, Norwich Research Park, University of East Anglia, Norwich, NR4 7TJ UK; 4 Norfolk & Norwich University Hospitals NHS Foundation Trust, Obstetrics & Gynaecology, Colney Lane, Norwich, NR4 7UY UK

**Keywords:** Endometriosis, Diagnostic delay, Diagnosis, Healthcare professional, Qualitative

## Abstract

**Background:**

Delay to diagnosis of endometriosis is an increasing problem. As it stands, the mean delay to diagnosis time is nine years. There is limited research exploring the perceptions of healthcare professionals regarding the diagnosis and respective delay to diagnosis of endometriosis in the United Kingdom. This study aims to explore this further.

**Methods:**

This study used an interpretive qualitative approach involving healthcare professional perspectives on reasons for delay to diagnosis of endometriosis. A series of focus group interviews with 15 healthcare professionals (General Practitioners (GP), gynaecologists and nurses) were conducted to explore their experiences of (the pathway to) diagnosing endometriosis. The data were analysed using reflexive thematic analysis.

**Results:**

Three main themes were identified: (1) masking and unmasking the symptoms, (2) power of the witness in diagnosis and (3) experiences that hinder the threshold to diagnosis. The presence of the patient alone is not always enough to facilitate a diagnosis, and as such, the presence of another individual, quite often a male partner enables the threshold to referral and diagnosis to be crossed.

**Conclusions:**

Healthcare professionals in this study described how endometriosis is often masked or rendered invisible, and how the presence of another person—most often a male partner—can legitimise symptom severity and influence referral decisions. Healthcare professionals should consider reflecting on how another individual in a consultation influences their thinking process regarding the diagnostic journey of an individual with suspected endometriosis. They should also consider their own preconceptions of endometriosis as an illness to explore how best they can support individuals with suspected endometriosis. These practical suggestions can be used to reduce the delay to diagnosis of endometriosis and bring positive change to the diagnostic process of people living with endometriosis.

**Clinical trial number:**

Not applicable.

## Background

Endometriosis is a common condition that is known to affect multiple systems and is a frequent cause for chronic pelvic pain in women [1]. The National Institute of Clinical Excellence (NICE) [2] have published guidelines on the diagnosis and management of endometriosis and made extensive reference to the timeframe involved in diagnosing women with endometriosis. One specific recommendation was to explore ways in which the diagnosis time for endometriosis can be reduced. The mean delay to diagnosis time in the United Kingdom is 9 years (Endometriosis UK) [3].

Endometriosis is suspected in individuals with, but not limited to, chronic pelvic pain, dysmenorrhoea, pain during sexual intercourse, cyclical gastrointestinal or urinary symptoms and/or infertility and [4]). Initially an ultrasound pelvis is recommended, or if deep infiltrating endometriosis is suspected, then an MRI (magnetic resonance imaging) scan may be appropriate (NICE, 2024) [2]. The updated NICE guidance [2] in 2024 makes a clear recommendation on performing a diagnostic laparoscopy to diagnose endometriosis, even if the ultrasound pelvis or MRI findings are normal. Investigations for suspected endometriosis and any respective referral to secondary care gynaecological services should occur in parallel (NICE, 2024) [2].

There is a small but growing body of research exploring the experiences of healthcare professionals in diagnosing endometriosis. One of the major challenges in diagnosing endometriosis is the healthcare professional’s ability to differentiate between ‘normal’ and ‘abnormal’ menstruation pain [5]. Through telephone interviews with 42 general practitioners, Dixon et al. [6] found that endometriosis can present with non-specific symptoms and this itself can hinder diagnosis. The variation in which pain related symptoms are communicated by patients to healthcare professionals can present challenges in recognising endometriosis as a differential diagnosis, particularly when metaphorical descriptions are used [7], for instance ‘a stabbing pain’. Limitations in expertise in the diagnosis of endometriosis within primary care has also been cited a challenge in diagnosing the condition [8] Rowe et al. 2021) [9]. Within the primary care context, a wide range of differential diagnoses need to be considered whilst also suspecting endometriosis as patients often present with undifferentiated symptoms that may be present in several other medical conditions [10].

Endometriosis impacts women’s quality of life in various ways disrupting work productivity with subsequent financial implications but also negatively impacting physical, psychological and sexual function [11]. Many women tolerate significant amounts of pain and heavy menstrual bleeding for many years in silence [12]. Moreover, impact on quality of life has been shown to be linked to time to diagnosis [12]. Endometriosis has a significant impact on psychological health [13] exacerbated by reported healthcare professional neglect leading to diagnosis and treatment delay. Hållstam et al. [14] concluded that healthcare professionals should be more mindful of the grief that is associated with living with this condition and find ways of supporting women through this grief.

Gaining a diagnosis allows the patient to understand and rationalise their symptoms and subsequently enables them to obtain treatment [15]. For example, for endometriosis, treatment (hormonal or surgical) has demonstrated a positive impact on the health-related quality of life [14]. Moreover, a diagnosis enables patients to legitimise their symptoms and subsequently have a medical explanation for them [16]. Finally, a diagnosis enables individuals to make informed decisions about their healthcare, whether this involves an intervention or not [17].

Delay to diagnosis of endometriosis is increasingly being recognised as a problem amongst researchers and the public domain. In 2020, the United Kingdom (UK) All-Party Parliamentary Group [18] on endometriosis found that delays to diagnosis continue, with an average diagnostic delay of 7.5 years from symptom onset to diagnosis; this time has since increased up to 10 years [19]. In addition, of the 10,000 participants who completed the APPG survey online, 58% of them visited their GP more than ten times prior to receiving a formal diagnosis (confirmed by imaging, laparoscopy or histology), 21% saw a hospital doctor more than ten times and 53% visited the accident and emergency (A&E) department. Recently, the UK government introduced the teaching of menstrual wellbeing for all pupils in primary and secondary schools in England [20]. However, education alone is unlikely to reduce delays in diagnosis [6], so it is likely that a combination of factors will influence diagnosis; therefore, there is a need to explore other ways to facilitate an earlier diagnosis.

Existing evidence indicates that women’s healthcare experiences of endometriosis diagnosis can be very challenging [5]. Further research is required to explore how healthcare professionals (general practitioners, gynaecologists and nurses) navigate through the process of endometriosis diagnosis and how this may influence the delay to diagnosis. The overarching research question is: what are the perceptions of healthcare professionals with regards to the endometriosis diagnostic pathway?

## Method

This was an interpretive qualitative study [21], using focus groups conducted with healthcare professionals: GPs (including registrar grade), primary care nurse, endometriosis nurse specialist, general gynaecology nurse, or gynaecologist (including registrar grade). Registrar grade refers to a doctor who is training to become a GP or consultant. Each focus group included five participants (either five GPs, five gynaecologists or five nurses) and each group interview lasted between 80 and 180 minutes. All participating healthcare professionals were involved in the care of patients with endometriosis. For clarity, the term ‘care’ was defined as ‘anyone who is involved in diagnosing or treating a patient with suspected or confirmed endometriosis’ in either primary or secondary care. Table 1 shows the participant demographic information that was collected via a questionnaire prior to the focus group interview commencing.

**Table 1:** Participant demographics

Healthcare professional type	Age	Sex	Pseudonym	Ethnicity	Time since qualifying as a healthcare professional (years)
Focus group 1					
General Practitioner (GP1)	58	Male	James	White British	26
General Practitioner (GP2)	56	Male	Carl	White British	22
General Practitioner (GP3)	34	Male	Richard	White British	8
General Practitioner Registrar (GP4)	32	Female	Stella	White British	4
General Practitioner Registrar (GP5)	28	Male	Kieron	Black African	4
Focus group 2					
Gynaecologist 1 (Consultant)	53	Male	Gopal	Asian Indian	22
Gynaecologist 2 (Consultant)	52	Male	Jordan	White British	16
Gynaecologist 3 (Consultant)	39	Female	Mamta	Asian Indian	12
Gynaecology 4 Registrar	37	Female	Holly	White British	8
Gynaecology 5 Registrar	31	Female	Rebecca	White British	5
Focus group 3					
Nurse 1 (Endometriosis Specialist)	55	Female	Lisa	White British	22
Nurse 2 (Gynaecology Sonographer)	33	Female	Olivia	White British	8
Nurse 3 (Hospital ward-based)	36	Female	Emma	White British	12
Nurse 4 (Hospital ward-based)	28	Female	Charlotte	White British	7
Nurse 5 (Primary Care)	42	Female	Priyanka	Asian Pakistani	16

This study forms part of a larger project where the first phase involved semi-structured interviews with participants with confirmed endometriosis [11] in a grounded theory study. To conduct the second phase of the study with healthcare professionals, the interview topic guide contained verbatim quotes from our previous study [11] to aid discussion. It was important to adopt a methodology that connected both phases of the project and this is drawn from an interpretive approach [21] and as such reflexive thematic analysis (RTA) [22] was used as both a method and methodology. Prior to deciding on RTA other approaches, such as phenomenology, grounded theory and framework analysis were considered. The reasons for choosing RTA are three-fold. Firstly, our epistemological position was that of a qualitative and interpretive approach; the way in which data are organised and interpreted through RTA is consistent with this. Secondly, approaches such as framework analysis appeared reductionist, in the sense that a framework is developed from an initial focus group and this ‘rigid structure’ is then applied to the subsequent transcripts, even if there is no clear applicability [23]. Finally, the research objective for the healthcare professional phase of the study was designed to focus on a central observation that involved the delay to diagnosis of endometriosis; an RTA approach enabled this to be explored and complement the grounded theory findings to answer the overarching research objective which was to understand delay to diagnosis.

### Data collection

The study team comprised of a registrar in obstetrics and gynaecology (BK), consultant gynaecologist (EM), health psychologist (JS) and academic nurse (GT). A combination of snowball and purposive sampling ensured that a broad range of individuals within each occupation (GPs, gynaecologists and nurses) were selected [24] and [25]). Focus groups interviews were conducted by BK to enable the respective healthcare professional group to explore and discuss their experiences of diagnosing endometriosis. The interviews were conducted in the gynaecology outpatient department for gynaecologist and nurse participants, and in a primary care setting for GP participants. BK attended several training sessions on conducting research interviews prior to the study commencing. Focus group interviews are increasingly used in healthcare research to generate a research narrative though group facilitation [26, 27]. This approach is often used to explore and understand meanings and processes and allows narratives among participants to be explored in detail [28]. Each focus group interview with the respective types of healthcare professionals were conducted separately to ensure power dynamics, if any, did not influence the interactions. In addition, each of the focus group interviews used the same semi-structured interview topic guide (Supplementary File 1). Prior to the commencement of each focus group interview, all participant consent forms were checked to ensure they had been signed, and it was also confirmed that the participants did not have any questions or concerns to ensure informed consent was obtained. All interviews and focus groups were audio recorded.

### Data analysis

After each focus group interview, the respective audio recording was transcribed verbatim by BK. All participants were assigned a pseudonym on transcription to ensure anonymity [29]. The data was analysed using the six phases of reflexive thematic analysis [30] by JS and BK. Familiarisation involved an initial listening to the audio recordings to make sense of them overall and the transcripts were read in detail at least twice to obtain a deeper understanding of the data. Initial codes were then developed and through this process initial themes were generated from the codes by exploring patterns amongst the data. Through an iterative process, the initial themes were then reviewed and refined at ‘two levels’: (1) reviewing the transcript extracts under each code and ensuring they fit within the particular theme and (2) the themes were explored within the wider dataset to ensure they were connected [30]. The final phase offered the opportunity to ensure that all the themes were connected and evidenced with the appropriate data from the focus group interviews to create the final report [30].

In qualitative research, the term ‘reflexivity’ is used to describe the impact a researcher has on the research process [31]. As a male registrar in obstetrics and gynaecology, BK was interviewed by an experienced qualitative researcher prior to the study to explore any unconscious pre-conceptions that might influence his stance during data collection and documented as written reflections (personal reflexivity) [32]. BK was aware his role as a healthcare professional may influence the focus group interviews and as such, made his position clear as a researcher only with participants. As a male researcher, with no personal experience of gynaecological illness, BK was actively able to clarify meaning during the focus groups, rather than make assumptions. JS and GT were advanced qualitative researchers compared to BK and EM; therefore, interpersonal reflexivity [33] was important through regular meetings with all team members to discuss and address the ongoing research process. The transcripts from the focus groups were analysed and re-analysed on several occasions by BK and JS. Through many discussions, methodological reflexivity was practised as we proceeded through the reflexive thematic analysis [31].

Research rigor was maintained through several ways: the focus group interview schedule was amended following several discussions amongst the team prior to its implementation, the interviews were transcribed verbatim immediately and the use of verbatim quotes from our previous study [11] enabled triangulation of data [34] to further understand the processes of endometriosis diagnosis.

The study was approved by the London-Surrey Borders Research Ethics Committee via the Integrated Research Application System (IRAS) (approval no. 223380) and conducted in accordance with the ethical principles outlined in the Declaration of Helsinki.

## Findings

After analysing the narratives using reflexive thematic analysis, three overarching themes were generated from the analysis. The first theme, *masking and unmasking the symptoms*, describes how endometriosis diagnosis delay has been created through masking in several different ways for healthcare professionals. The second theme, *power of the witness in diagnosis*, reflects the discovery that the woman’s pathway to a diagnosis is often (required to be) facilitated by another person in order to be able to get across the threshold to get the referral she needs. The third theme, *experiences that hinder the threshold to diagnosis*, discusses the various presumptions held by healthcare professionals that has an influence on the diagnostic journey, and as such, the delays to diagnosis of endometriosis.

## Theme 1: Masking and unmasking the symptoms

This theme contains three sub-themes: ‘hidden nature of (female) pain’, ‘masking through symptom mimicry’, ‘masking through ultrasound scan reporting procedures’ and ‘unmasking through the correct referral process’.

### Hidden nature of (female) pain

Healthcare professionals discussed the challenges of interpreting pain symptoms and how this can make the diagnosis challenging. The hidden nature of endometriosis, particularly in relation to the impact the symptoms can have on a woman’s life was expressed:

‘It really is a hidden illness. How can you quantify pain?! How can you*understand the sexual dysfunction? How can you understand the* *psychosocial impact it can have? It’s all a hidden mystery unless you* *ask’’ (Holly, gynaecologist 4)*

The reporting of pain was described as difficult for healthcare professionals to respond effectively to:

“Pain is a common symptom for women with suspected endometriosis. You can’t visualise pain and so it makes it difficult to appreciate the severity of it and so it doesn’t make you want to refer to the hospital straight away”. (James, GP 1)

One gynaecologist discussed how recognising and differentiating normal menstruation from abnormal menstruation is a key part of making the diagnosis of endometriosis:

“How can you just differentiate between normal and abnormal [menstruation] especially in the community? I know that GPs will try their best to differentiate between what patients need to come to the hospital and what patients can stay there, but it’s hard. With endometriosis, I suppose a big part of what is normal and abnormal will depend on the patient’s history and maybe the persistence of symptoms or lack of improvement in symptoms?” (Rebecca, gynaecologist 5)

Despite this knowledge gap, the GP training programme does not always incorporate women’s health as part of training:

‘And so, a load of trainees do not get specialty training in gynaecology. They can go straight through GP training with no exposure at foundation level or GP training! Interpretation of symptoms is therefore hard for them’ (Stella, GP 4)

### Masking through symptom mimicry

The diagnosis of other common medical conditions that may mimic the symptoms of endometriosis can mask a diagnosis; one healthcare professional commented that in the presence of a diagnostic laparoscopy that has not detected endometriosis by the gynaecologist, conditions such as irritable bowel syndrome are often diagnosed instead:

“To be fair, even general gynaecologists will label someone as IBS if the laparoscopy is normal. It’s certainly not on the Rome criteria to make a diagnosis of IBS by laparoscopy! I have seen this a number of times” *laughs* (Carl, GP 2)

The wide range of other differential diagnoses that need to be considered by the healthcare professional can make endometriosis as a consideration challenging:

“There are so many differentials to think about- urinary tract infection, fibroids, irritable bowel syndrome etc. This makes it hard to think about endometriosis as a top differential” (Kieron, GP 5)

Symptoms of endometriosis are normalised when the healthcare professional is presented with unexplained symptoms:

‘You might be surprised how many women will have symptoms that settle with what we do in primary care. However, sometimes a cause for symptoms is not always obvious and it is easy to normalise it as a doctor’ (Richard, GP 3)

### Masking through ultrasound scan reporting procedures

A dual nurse/sonographer participant discusses that despite reporting a normal ultrasound scan, she perceives the reporting system as not encouraging her to retain endometriosis as a consideration. She shares how this will result in a fragmented patient journey towards diagnosis:

“When I scan people, I sometimes think “this woman most probably has endometriosis”. However, the ultrasound might be normal, but when you ask the woman more questions about symptoms, she has clear symptoms of endometriosis. As a nurse sonographer, I can’t do anything with this information unfortunately. I can just write on the report: normal scan. Then you can see the patient getting bounced back” (Olivia, nurse 2)

### Unmasking through the correct referral process

Healthcare professionals discussed the benefits of a specialist gynaecologist with training in endometriosis care (such as that accredited by the British Society of Gynaecological Endoscopy, BSGE) and how this might compare to a generalist gynaecologist without specific endometriosis surgery training:

‘”Obviously a specialist in the area is going to be able to provide‘better care’ compared to a general gynaecologist. Every gynaecologist should be trained in some element of endometriosis surgery” (Gopal, gynaecologist 1)

“There is often more collaborative care with an endometriosis centre with urology and colorectal teams. There will of course be access to an endometriosis nurse specialist who is a contact point for patients’ (Rebecca, gynaecologist 5)

One healthcare professional discusses how the healthcare professional must be mindful about which type of trained gynaecologist is appropriate as part of a referral, and those with suspected bowel, bladder or distant involvement (diaphragmatic) should be referred to a specialist centre:

*“I think that I have encountered women who have been seen by a general gynaecologist and a specialist centre. I think there is a marked difference between the two. With the specialist centre, it is perceived as providing a higher level of care compared to a general gynaecologist or district general for severe disease” (Carl, GP 2)*

## Theme 2: power of the witness in diagnosis

Healthcare professionals discussed the ways in which individuals with suspected endometriosis attempt to project their visibility to be heard in a clinical consultation through the influence of another individual, a witness to her symptoms, who was quite often a male partner. The influence of another person in a consultation was significant and this impacted on the healthcare professionals’ perception of the severity of the patients’ symptoms, and this frequently led to further investigation(s). This theme is underpinned by two subthemes: increasing visibility through another person and the impact of another healthcare professionals’ voice.

### Increasing visibility through another person

Many of the healthcare professionals in the study discussed the influence of a significant other (most commonly a male partner) in a consultation and how their presence adds weight to the process of understanding and listening. His voice in the consult carries, it seems, *more* weight – either by adding to the woman’s lone voice or by carrying more weight than the woman’s voice entirely:

“It [presence of another person in a consultation] adds weight and gravity to the psychosocial impact of the condition. If a patient is telling you this, but their partner is also saying this, then it makes you think again. It might be that the partner is telling you that they are not having sex anymore and it certainly adds more weight to the psychosocial aspect” (Richard, GP 3)

For some, the mere presence of another person is a powerful signal to the healthcare professional that a referral should be considered:

“I would definitely think twice about not referring someone if another person is present. It would make me question why someone else is also present and so listen differently” (Carl, GP 2)

Healthcare professionals discussed how It is also helpful to have someone else (a romantic partner for example) in the consulting room as it helps the woman with suspected endometriosis to triangulate her symptoms of endometriosis:

“If you were the patient, it would be hard to remember everything you want to get across. Therefore, the person you are with in the room, they are often detached and so they can get across points maybe you might forget as the patient. It alters the dynamic and it does it in an effective way. It allows you to get the history in a different and dynamic way with another person” (Stella, GP 4)

“Oh yes, I see this all the time. It’s quite usual for a woman to bring a friend or her partner into the clinic with them. This is a really good thing. If the woman has not taken in everything we have discussed, then it’s quite likely that the other person will have remembered. This is nothing but good” (Mamta, gynaecologist 3)

Although this was not always experienced as something positive especially when the voice of the other person was seen to take away from the voice of the patient:

“Yes, that’s true. I have had occasions where this has been the opposite. The partners of some patients have been quite challenging and tried to almost take over the consultation. I have had to tell them to politely to back off. Maybe this is because they are also frustrated on the patient’s behalf and just want to make sure their voice is heard” (Jordan, gynaecologist 2)

### The impact of another healthcare professionals’ voice

Individuals with suspected endometriosis sometimes attend A&E with written documentation from their GP medical records that indicate a diagnosis of endometriosis is suspected. This evidence in the form of a medical letter demonstrates the value of one healthcare professional communicating with another:

“It’s interesting that this woman has felt the need to take her discharge letters with her to A&E. I wonder what this represents. Surely A&E have records of this, or the patient is able to tell them anyway. I think a lot of women are stuck when they go to A&E, and equally, healthcare workers in A&E must also be stuck- apart from offering more pain relief or calling the gynaecology SHO [senior house officer- doctor in training] for advice. Here, the women think that taking her discharge letters in with her may make the clinician listen to her” (Carl, GP 2)

## Theme 3:experiences that hinder the threshold to diagnosis

Healthcare professionals highlight a number of challenges about the way endometriosis is diagnosed and contributes to the delay in diagnosis of the condition. This theme contains three sub-themes: (1) challenges relating to primary care, (2), generalist versus specialist gynaecologist and (3) interpretations of the term ‘chronic pelvic pain’.

### Challenges relating to primary care

If an inadequate medical history is taken in the primary care setting, then endometriosis may not be suspected in a timely manner, and this may influence the time taken to refer the patient to a gynaecologist or for any imaging procedures.

‘Patients often say the pain is worse on their periods and when they open their bowel. However, the GP referral might say something very different! It might say “chronic pelvic pain, ?cyst” on the scan referral. This happens a lot from GPs’. (Olivia, nurse 2)

Gynaecologists discussed the influence of prior negative healthcare experiences on the expectations that the patient has with them.

“Even when I run the endometriosis clinic with a consultant, patients have huge expectations [to be diagnosed] when they see you. They have often had negative experiences with healthcare workers before seeing us” (Holly, gynaecologist 4)

“As an endometriosis nurse, some patients have told me they encourage other women with suspected endometriosis to ask the GP for a direct referral to a gynaecologist due to their own bad experience. This isn’t helpful as it implies that GPs are not capable of diagnosing endometriosis’ (Lisa, nurse 1)

A healthcare professional provided insight into the challenges that GPs may face in primary care when first encountering patients with suspected endometriosis. One clinician discussed how a ten-minute appointment is a limited time period and speculated that this can impact on women’s healthcare experiences.

‘I understand that it’s quite difficult to ascertain all the concerns in a 10-minute appointment. Maybe these patients need longer in the GP clinic?’ (Mamta, gynaecologist 3)

### Generalist versus specialist gynaecologist

The differences between a general gynaecologist and a gynaecologist specialised in endometriosis was discussed by the nurse participants in particular. They perceived that generalist gynaecologists will ‘not want to get involved’ and will want to refer the patient to an endometriosis specialist.

‘I wonder if the general gynaecologists are quite keen to get the patient to the endo specialist and so they don’t want to get involved in their care too much?’ (nurse 2)

Gynaecologists themselves also discussed how they perceived differences between generalist and specialist gynaecologists.

‘I always seem to hear the same story from patients. Some GPs are either very good at following up on these patient concerns and some not. When I see referrals from another hospital, it is often because the patient is not happy with the team. There is definitely a difference between a general gynaecologist and one that is an endo specialist’. (Gopal, gynaecologist 1)

One healthcare professional was under the assumption that a generalist gynaecologist may not want to perform a diagnostic laparoscopy for suspected endometriosis but may consider hormonal treatment only in the first instance.

‘I think gynaecologists must be presented with so many women with the expectation that they need to have a laparoscopy. Therefore, they will try and treat the symptoms without having to do the invasive procedures’. (Carl, GP 2)

A more specific way this difference is expressed is in the management options that are recommended to the patient. For instance, hormonal treatment with a gonadotrophin releasing hormone (GnRH) analogue is sometimes considered for those patients with endometriosis in whom removal of the ovaries is being considered. However, it appears that the use of this drug without a diagnosis of endometriosis can do more harm than good, and as such may even delay the diagnosis:

“The problem I have is that I have referred patients to a local gynaecologist, but instead of a diagnosis, the gynaecologist has whacked them on a load of GnRH analogue, which is a horrendous drug and to induce the menopause, without getting a diagnosis. Just because they don’t want to do a laparoscopy. Once people know how good an endometriosis specialist centre is and know they are going to get a good deal, you will get a shed load of referrals” (James, GP 1)

### Interpretations of the term ‘chronic pelvic pain’

The term chronic pelvic pain is a non-specific term, and participants highlighted there may be differences in the interpretation of this term. The use of the term ‘chronic pelvic pain’ was discussed by one healthcare professional where they explain that using these words when an underlying cause has not been found may be damaging:

“Also, if the patient has been labelled with chronic pelvic pain, but without a diagnosis, then surely this has an influence on the perception of A&E clinicians towards these patients?” (Kieron, gynaecologist 5)

Another healthcare professional explains that the use of the label ‘chronic pelvic pain’ in the GP medical records may mean the woman’s symptoms may be dismissed in an Accident and Emergency (A&E) setting:

“To me chronic pelvic pain means that no cause has been found and therefore A&E staff may dismiss the pain if the woman presents with pain symptoms if its on the GP summary record” (James, GP 1)

## Discussion

Our study has explored healthcare professionals’ perceptions on the process of and delays to diagnosis of endometriosis using direct verbatim extracts from women diagnosed with endometriosis from our connected study that is associated with a wider project [11]. Our findings demonstrate that the diagnosis of endometriosis is a puzzle that requires unmasking, involves a multitude of individuals and necessitates assumptions to be overcome. The way in which endometriosis presents clinically can sometimes be through vague and nonspecific symptoms, and as such, can make suspecting endometriosis a challenge. As a result, endometriosis is masked whilst other medical conditions mask endometriosis related symptoms. The presence of the patient alone is not always enough to facilitate a diagnosis, and as such, the presence of another individual, quite often a male partner enables the threshold to referral and diagnosis to be crossed. The power of another individual in the consulting room, either in person or through medical documentation was resonant throughout the findings. Several assumptions made by healthcare professionals about each other can hinder further exploration of the symptoms, and as such, the diagnosis. The disconnect between the primary and secondary care interface results in delays to diagnosis.

## Findings in relation to the wider literature

Most of the evidence exploring women’s experiences of being diagnosed with endometriosis is from secondary care data and as such, does not always reflect those women who are managed in primary care [35]. Our study has explored the experiences of several healthcare professional groups within primary and secondary care to obtain a broad view of the delays to diagnosis. It is also important to be mindful that from a primary care perspective, women with undiagnosed endometriosis present with undifferentiated symptoms, and as such, the diagnosis of endometriosis must be considered within the context of other medical conditions [36]. There are several studies that state awareness of endometriosis amongst GPs must increase [35–40]. Whilst this is important, Dixon et al. [6] found through focus groups with GPs that simply having an awareness of endometriosis amongst healthcare professionals in not enough to diagnose the condition; our findings also corroborate this. It is also clear that the non-specific symptoms associated with endometriosis, as well as undifferentiated presentations encountered by GPs, means that a series of differential diagnoses need to be considered, including suspected endometriosis [10]). The general nature of the training undertaken by GPs places them in a unique position to consider endometriosis as a differential diagnosis [10].

The impact of another person in the consultation is discussed in our study where the presence of another individual will influence the perception of the healthcare professional. Several studies have found the influence of a ‘companion’ in a medical consultation to be positive [41, 42]). However, further research is required in this area with regards to endometriosis to help healthcare professionals navigate successfully through this triadic consultation with another person also present in the consultation. The presence of a male partner in the consultation facilitating a diagnosis of endometriosis reflects the patriarchal medical system in which medicine is practised [43]. We are able to understand the complexities of patient experiences and the societal influences that impact on the delay to diagnosis. Our findings challenge us to not only explore how healthcare professionals navigate through to a diagnosis of endometriosis, but to also consider how societal biases influence the diagnostic journey. Several studies have explored the positive aspects of having another person in a medical consultation [41, 42]) and whilst our research has found how healthcare professionals recognise and respond to the influence of a male partner in a consultation, further research is required to explore how these impacts on the clinician’s decision-making process when diagnosing endometriosis and the influence of this on the woman’s care experience and whether her voice is heard or not.

The factors associated with the delays to diagnosis of endometriosis are complex and intertwined Dixon et al [44]. Both patients with suspected endometriosis and healthcare professionals alike tend to normalise symptoms before they are considered abnormal [45]. Normalisation of abnormal symptoms is dangerous, the least impact of which is delay to diagnosis of endometriosis [46]. In keeping with our findings, Fernley [45] found that the normalisation of pain and symptom dismissal by healthcare professionals contributed to the delay in diagnosis of endometriosis.

Healthcare professionals hold power through the act of diagnosing, prescribing, referring to specialties, and treating [47]; this was discussed by healthcare professionals in our study. In the medical context, it is important for healthcare professionals to appreciate the power that they hold through their role as the gatekeepers to secondary care. However, there is limited research into how healthcare professionals themselves recognise and navigate through the power dynamics during a medical consultation. The perceived power dynamic between a patient and healthcare professional has been recognised in many studies [48–50]. Power comes in many forms and is seen in many different contexts: marriage, employment, the law, between a child and parent, and in the medical context between a patient and healthcare professional [50]. One way in which healthcare professionals may navigate through perceived power imbalances with a patient is through shared decision-making [51]. Shared decision-making is an approach to the medical consultation which allows the power to be distributed equally between the patient and healthcare professional and enable the patient to make better sense of their symptoms [51]. In the case of endometriosis, through shared decision-making a healthcare professional is able to make a better assessment of the information provided by the woman, with a view to helping her understand the various options that are available to explore her unexplained symptoms.

Nimmon and Stenfors-Hayes [47] explored healthcare professionals’ perceptions of power in the clinical encounter and found that healthcare professionals do not always recognise the influence of power dynamics on shared decision making in these circumstances and therefore do not know how to address them. In addition, the importance of healthcare professionals recognising how their medical practice may influence a woman’s participation in a consultation has also been recognised [52]. It is therefore vital that healthcare professionals reflect on their own clinical encounters, recognise any elements of power imbalance and understand how this can influence women’s health-seeking behaviours. This reflection could be part of continuous professional development and by attending standardised diversity training in healthcare.

Endometriosis related symptoms can be vague and therefore diagnosis can be challenging, as discussed by healthcare professionals in our study. Rasmussen and Ro [53] found that in patients with “medically unexplained symptoms” if a biopsychosocial approach is utilised during a consultation, more information is likely to be gathered from the patients and result in a diagnosis due to shared decision making. Numerous research studies [54, 55], including our findings, show that shared decision making between healthcare professionals and patients with endometriosis does not always occur. A pivotal systematic review by Dancet et al. [56] found that patients with endometriosis value a medical consultation that is patient cantered as well as the ‘involvement of significant others’.

## Strengths and limitations

Our research is unique in that it does not focus on healthcare professionals but rather healthcare professionals’ perceptions of the diagnostic process. The findings relating to the perception of healthcare professionals of another person in a medical consultation (namely partners) is unique; this information furthers our understanding of diagnostic delay amongst those with endometriosis. The inclusion of a variety of healthcare professionals, instead of only medical professionals, is a strength. Our study design incorporated the findings from our other connected study [11] through verbatim quotes; this is a novel and unique methodological contribution in advancing our understanding of endometriosis diagnosis delays.

As a limitation, the findings from our study must be considered within the context that all the GPs who participated in the focus group study all worked at a single practice in the East of England, so the views of GPs who work at other practices have not been considered. Single discipline discussions did not create a place for debate or discussion amongst the different healthcare professionals. Other professionals, including Accident and Emergency medicine, pelvic physiotherapists and sonographers could have been included; after reading the wider literature and interviewing healthcare professionals, it became clear this group of professionals also have a role in recognising endometriosis as a diagnosis.

## Recommendations for policy and practice

The recommendations for policy and practice presented in this article must be applied within the complexities of an ever-changing healthcare system in the UK. A truly connected multidisciplinary approach between primary care and secondary care services is required to enable the voice of all healthcare professionals to be heard and therefore facilitate the diagnosis process [10]. Healthcare professionals should consider reflecting on how another individual in a consultation influences their thinking process regarding the diagnostic journey of an individual with suspected endometriosis. Healthcare professionals should also consider their own preconceptions of endometriosis as an illness and explore how best they can support individuals with suspected endometriosis. Whilst only one participant discussed this, for those patients suspected to have bowel, bladder or distant site endometriosis should be promptly referred to a BSGE centre for specialist input in the diagnosis and management of endometriosis (NICE, 2024) [2].

Menstrual wellbeing and gynaecological health education for healthcare professionals should be further incorporated into the existing GP training programme and for allied health professionals working within the primary care setting (advanced nurse practitioners for example). When endometriosis is suspected, discussing the potential pathway to diagnosis can offer patients insight into what to expect and as such, create a more positive journey to diagnosis. The individual affected by symptoms of suspected endometriosis should be empowered to know what is ‘normal’ for her in terms of her menstrual wellbeing and crucially, what is not; healthcare professionals are in a unique position to support with this.

## Future research

The climate of primary care has evolved over the years. Increasingly, there are a range of professionals that work within the system, to include advanced nurse practitioners, pharmacists and paramedics within General Practice. Further research should explore their experiences of managing women’s health within their scope of practice and identify where further support may be required to minimise the delay to diagnosis. The role of another person, apart from the patient, within the medical consultation is important; future research should explore their experiences as the advocate and how this can facilitate diagnosis as well as how women with endometriosis themselves perceive and understand this. Further, improving the experiences of women’s journey to diagnosis remains an imperative and research designed to address this should be strongly encouraged.

## Conclusions

Delays in diagnosing endometriosis are shaped by clinical complexity, assumptions about women’s health, and relational dynamics within consultations. Healthcare professionals in this study described how endometriosis is often masked or rendered invisible, and how the presence of another person—most often a male partner—can legitimise symptom severity and influence referral decisions. Improving timely diagnosis requires greater interprofessional collaboration, reflexivity about power in clinical encounters, and more inclusive, patient-centred care.

## Data Availability

The datasets used and/or analysed during the current study are available from the corresponding author on reasonable request.
